# What we learned from extended culture of ‘rejected’ day-3 cleavage stage embryos: a prospective cohort study

**DOI:** 10.1186/s13048-017-0332-5

**Published:** 2017-05-16

**Authors:** Anat Hershko Klement, Michal Ovadia, Amir Wiser, Arie Berkovitz, Tal Shavit, Luba Nemerovsky, Yehudith Ghetler, Ilan Cohen, Adrian Shulman

**Affiliations:** 1 Department of Obstetrics and Gynecology, IVF Unit, Meir Medical Center, 59 Tchernichovsky St, Kfar Sava, Israel; 2 Sackler Faculty of Medicine, University of Tel Aviv, Tel Aviv, Israel

**Keywords:** Embryo transfer, Embryo culture techniques, Blastocyst, Embryo implantation

## Abstract

**Background:**

To test whether poor quality day-3 embryos can undergo successful blastulation and implantation.

**Methods:**

A prospective cohort study was conducted. Whether or not a good quality embryo was transferred on day-3, poor quality (rejected) embryos were further cultured and followed. The clinical outcome of each embryo was assessed.

**Results:**

A total of 694 rejected embryos (from 205 patients) were included, with a blastulation rate of 21.2% (147 embryos) compared to 64.2% general blastulation rate reported by our laboratory (*P* < 0.01). In a multivariate logistic regression model, only their grade on day-3 significantly affected blastulation (*P* = 0.01). A total of 97 embryos attained eligibility for fresh transfer or cryopreservation, only 6 of which resulted from a day-3 embryo scored < 2. Of these, 52 were transferred, resulting in 21 pregnancies (16 clinical and 5 chemical). In summary, 694 cultured embryos yielded 16 clinical pregnancies; a 2.3% clinical pregnancy rate.

**Conclusions:**

Low score day-3 embryos can result in successful blastulation and clinical pregnancies. However, the normal blastulation rate is poor.

## Background

Embryo morphology correlates with successful implantation [1–5]. Several aspects of embryo morphology before freezing, including cell stage, cell symmetry, cleavage pattern, synchronous versus asynchronous cleavage and percentage of anucleate fragments were found to influence post-thaw survival and implantation [6]. Embryologists and clinicians are uncomfortable when faced with decisions involving ongoing culture of poor-quality, day-3 embryos, as the true potential of a low-scoring embryo to yield a viable fetus is uncertain. This question is even more troubling when a given cycle yields only poor grade embryos. On the other hand, discarding an embryo, which has potential for future pregnancy, is certainly undesirable. Therefore, we evaluated whether these low-scoring day-3 embryos could lead to successful blastulation and implantation.

## Methods

This prospective cohort study was performed at a university-affiliated IVF unit (Meir Medical Center; Tel Aviv University, Israel). The study was approved by the Institutional Ethics Committee (0072-16MMC).

Morphology was scored by a team of 3 embryologists who have been working together for more than 10 years. Scoring is performed using 200–400× magnification on an inverted microscope. Scoring criteria in terms of day-3 morphological grading are grade 4 = equal sized, symmetrical blastomers and/or <10% fragmentation; grade 3 = uneven blastomers and/or 10–20% fragmentation; grade 2 = uneven blastomers and/or >20–40% blastomeric fragmentation and grade 1 = uneven blastomers and/or >40% blastomeric fragmentation.

The study was conducted from January 2012 to January 2015. The inclusion criteria for the embryos involved were as follows: before 2013 we included embryos that were not transferred on day-3 due to poor quality, but were followed until day-5. From 2013 on, all embryos morphologically graded **≤**2 on day-3 and cultured in a time lapse incubator as part of the routine algorithm were included. These embryos were followed up to day-6 and were either used for a fresh transfer/ cryopreservation or discarded (<2BB Gardner score) [7].

All cycles ending in embryos graded **≤**2 (termed “rejected embryos”) that were not transferred, but further cultured were included in the analysis. Rejected embryos were available when a good quality embryo (>grade 2) was transferred on day-3, but all other embryos were graded poor quality and further cultured, or when all embryos were graded <2 on day-3 and none were transferred at that time. In these circumstances, our laboratory policy requires further culturing until day-6 post-oocyte retrieval.

Each embryo was tested for the following parameters: day-5 fresh embryo transfer (day-6 embryos were not transferred, but frozen), freezing procedure, future thawing, transfer and clinical outcomes. Embryos that originated from a low-scoring day-3 embryo and were transferred along with those originating from a high grade day-3 embryo, were excluded, because the outcome cannot be attributed with certainty to the study embryos. When two blastocysts originating from rejected embryos were transferred together, they were counted as a single case in terms of pregnancy outcome.

### Statistical analysis

**Fig. 1:**
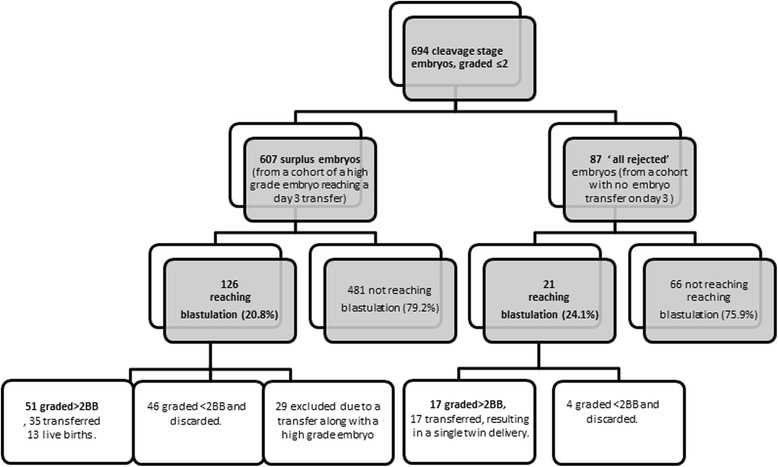
Flow diagram of the study population

The analysis was divided into 3 steps: step 1 to the point of blastulation (Fig. 1), step 2 to the point of a blastocyst scoring >2BB Gardner score and step 3 for correction within subjects. All calculations were performed using SPSS 23.0 (SPSS Inc., Chicago, IL). Normally distributed data were analyzed using unpaired student t-test. Chi-square or Fisher’s exact test were used for comparing rates and proportions. Data were correlated at the patient level; therefore, analysis of successful blastulation was performed using generalized estimating equation regression model, with a logit link function. An exchangeable covariance structure was used for the working matrix (patient as the subject variable, i.e. random). All *P*-values were tested as two-tailed and considered significant at <0.05.

## Results

A total of 694 rejected embryos (from 205 patients) were included in the study (Fig. 1). Of these, 147 embryos blastulated (21.2%), compared to a mean blastulation rate of 64.2% reported by our laboratory (*P* < 0.01). Of these, 318 (45.8%) were graded 1 and 376 (54.2%) were graded 2. Mean maternal age was comparable between embryos ending in successful blastulation and those arresting (Table 1). Mean day-3 score of embryos reaching the blastocyst stage and the presence of a maternal medical diagnosis in addition to infertility (such as thyroid disorder, glucose intolerance) were significantly related to embryo arrest (Table 1). Additional variables tested in univariate analysis (post-wash total motile sperm count, body mass index (BMI), infertility duration, day-3 FSH and smoking) were not significantly related to blastulation. Although, infertility duration was close to significant (*P* = 0.06). Ethnicity, indication for treatment and protocol type were not significantly different between groups. In a multi-variate, stepwise, conditional, logistic regression model, only day-3 grade significantly affected blastulation: for each additional point on day-3 score in our cohort, the chances for blastulation increased 2.7-fold (95% CI: 1.5–5.1, *P* < 0.01).

**Table 1:** Clinical characteristics of study population presented by blastulation success

Variable	Reached blastulation		*P*-value
	Yes	No	
Maternal age, years (mean ± SD)	31.6 ± 5.7	31.2 ± 5.6	0.4
Maternal BMI (mean ± SD)	24.7 ± 4.3	24.4 ± 4.5	0.6
Maternal smoking	13.3%	9.7%	0.4
Maternal additional medical condition	5.0%	16.4%	0.03
Day-3 FSH, IU/L (mean ± SD)	6.6 ± 3.1	6.9 ± 2.9	0.3
Post-wash total motile sperm count, millions (mean ± SD)	4.9 ± 5.6	5.4 ± 9.4	0.5
Infertility duration, years (mean ± SD)	2.3 ± 1.8	2.7 ± 2.0	0.06
Embryo day-3 grade, (mean ± SD)	1.73 ± 0.4	1.49 ± 0.5	<0.01

*SD* standard deviation

In the next step of the analysis, involving 147 blastocysts from 93 patients, all cases of blastocysts graded less than Gardner 2BB (50 embryos) were excluded. Among the remaining 97 blastocysts graded >2BB (Fig. 1), only 6 of originated from a day-3 embryo scored <2. None of the demographic parameters listed above were predictive of blastocyst eligibility for transfer. After excluding blastocysts that were transferred along with an embryo originating from high day-3 score (29 cases, Fig. 1), 68 blastocysts were available for outcome analysis. Of these, 52 were transferred (41 fresh and 11 thawed). Tested by their inclusion criteria, 17 (32.7%) of these embryos were from a cohort that were all rejected by day-3 and 35 (67.3%) were from embryos followed after a good quality embryo was transferred on day-3(Fig. 1). An additional 3 embryos were thawed, but did not survive the process and 13 were still frozen at the time of the analysis. Interestingly, we could not find a satisfactory association between the Gardner blastocyst score and the day-3 time lapse score (Chi-square for trend, *P* = 0.6).

The cumulative results to this point are 17 gestational sacs, which translates into 32.7% implantation rate, and 30.8% clinical pregnancy rate (a single case of twin pregnancy resulting from a double transfer of 2 rejected embryos). Among the 16 clinical pregnancies, 14 ended in live births (13 singletons, 1 set of twins) and 2 in first trimester miscarriage. One clinical pregnancy was the result of a day-3 embryo which was morphologically scored 1, while 4 pregnancies were the result of the lowest morphokinetic score in our system(<1). In addition, 5 chemical pregnancies were recorded. Only one clinical pregnancy resulted from thawed embryo transfer; the rest resulted from fresh transfers.

In summary, 694 cultured embryos yielded 16 clinical pregnancies, or a 2.3% clinical pregnancy rate. The last step of the analysis included a generalized estimating equation corrected for the patient level, which again supported the grade as the single significant factor influencing blastulation in rejected embryos (*P* = 0.01).

## Discussion

Embryo scoring is based on the rationale that the likelihood of successful implantation differs among embryos and that this is predictable, to a degree, upon their performance. We assume that microscopic or morphokinetic behavior of an embryo is related to its chromosomal milieu. Obviously, sometimes an embryo with a high score fails to implant, as may an unfavorable embryo result in a viable pregnancy. In an era where many embryologists are shifting toward day-5 or 6 embryo transfer (blastocyst transfer), some practitioners still choose to transfer day-3 embryos [8, 9]. Therefore, it is important to try to determine whether rejected embryos have a chance for a live birth. Even with a strict day 5 transfer policy, there might be limited capacity for culture, and the ability to estimate which embryos will arrest provides valuable knowledge.

Many studies support a strong association between embryo quality and the chances for successful implantation and eventual live birth [2–5, 10]. To the best of our knowledge, only a few groups reported their experience with the ongoing culture of poor-quality day-3 embryos. In 2001, Balaban et al. [11] questioned the feasibility of blastocyst stage embryo transfer in patients with only fair and poor quality cleavage-stage embryos on day-3. The group compared 158, day-5 embryo transfers originating from poor quality cleavage-stage embryos to a control group of 162, day-3 transfer cycles performed with embryos of similar quality. They reported a non-significant difference in the clinical pregnancy rate per transfer (27.2% and 33.5%, respectively, *P* > .05), but the number of embryos transferred is not acceptable in current practice. In the day-3 transfer group, a mean of 5.2 embryos were replaced per patient. A Chinese group [12] supported the ongoing culture of poor cleavage-stage embryos: 569 poor-quality embryos cultured in vitro formed 248 blastocysts, of which 138 were high-quality (24.25%). In agreement with the Chinese group, in 2016 Capodanno et al. [13] summarized the outcome of surplus cleavage-stage embryos that were thawed and transferred: the poor-grade embryos demonstrated the same implantation potential when compared to those with high scores. Nevertheless, when implantation occurred, embryo quality reflected a correlation to the chance for an ongoing pregnancy. Capodanno et al. suggested that even greatly damaged thawed embryos, should be transferred.

Our study demonstrated a low yield from 694 rejected day-3 embryos: 21.2% blastulated and only 2.3% ended in clinical pregnancy. The chance for blastulation was strongly correlated to embryo grading, supporting the correlation between embryo grading and outcome. Based on our data, we could not predict whether a blastocyst would reach transfer once it demonstrated ongoing cell division. The initial grade was not reflected at this point, probably because only a few grade 1, day-3 cleavage embryos did not arrest. Polycystic ovarian syndrome (PCOS) was previously suggested as a factor affecting egg competence and therefore, a possible factor leading to ART in some of these patients [14]. The 10.7% prevalence of PCOS among our population is low compared to other IVF centers (up to 30%). Therefore, it is unlikely that PCOS alone could explain the ‘rejected’ embryo phenomenon.

Are these figures enough to dictate policy? We do not think so, at least not at this point. Discarding an embryo is a crucial decision that should be based on the most complete information available. We do believe that in high-load units, with limited space, our data support a tentative policy of discarding the lowest scoring day-3 embryos. For facilities that enable ongoing culture, we have shown that live births can result from these rejected day-3 embryos. For patients faced with only poor-quality day-3 embryos, we encourage ongoing culture.

This study is limited by its descriptive nature and by departmental policy: we did not randomize the rejected embryos for culture versus transfer and generally, no poor-quality embryos (day-3 or day-5) were transferred. It did not include a control group to test the outcomes of poor-quality, day-3 transfers, but as shown in previous studies, day-5 embryos should probably be transferred in this scenario and the selection provided by blastocyst culture is safer.

Despite its descriptive nature, this study can assist physicians, embryologists and patients in resolving an everyday dilemma of an unfavorable embryo score. We also want to point out that morphological scoring is subject to inter-observer variability, but at least currently, time-lapse data do not seem to improve the decision-making process in these circumstances.

## Conclusions

We conclude that low scoring day-3 embryos that are not considered transferable can still result in successful blastulation and a live birth. Despite this, the cycle yield is poor in cases involving the lowest morphological score on day-3 and in cycles where all embryos receive the lowest morphological score. In light of the significant costs involved in embryo culture, ongoing culture of these latter cases seems questionable. We are reassured, that the transfer of a poor-quality embryo that results in pregnancy is not correlated with compromised perinatal outcome [2]. This is indeed a very important point for reproduction specialists and for concerned patients.
